# Delays in completion and results reporting of clinical trials under the Paediatric Regulation in the European Union: A cohort study

**DOI:** 10.1371/journal.pmed.1002520

**Published:** 2018-03-01

**Authors:** Thomas J. Hwang, Paolo A. Tomasi, Florence T. Bourgeois

**Affiliations:** 1 Computational Health Informatics Program, Boston Children’s Hospital, Boston, Massachusetts, United States of America; 2 Center for Pediatric Therapeutics and Regulatory Science, Department of Medicine, Boston Children’s Hospital, Boston, Massachusetts, United States of America; 3 Program on Regulation, Therapeutics, and Law, Division of Pharmacoepidemiology and Pharmacoeconomics, Department of Medicine, Brigham and Women’s Hospital, Boston, Massachusetts, United States of America; 4 European Medicines Agency, London, United Kingdom; 5 Department of Pediatrics, Harvard Medical School, Boston, Massachusetts, United States of America; Edinburgh University, UNITED KINGDOM

## Abstract

**Background:**

Few medicines have been approved for children, leading to rates of off-label prescribing reported to be as high as 90%. In 2007, the European Union adopted the Paediatric Regulation, which mandates that pharmaceutical companies conduct paediatric studies for all new medicines, unless granted a waiver. We aimed to evaluate the availability of paediatric trial results from studies required under the Paediatric Regulation for new medicines authorised in the EU.

**Methods and findings:**

The European Medicines Agency (EMA) public database of paediatric investigation plans was searched for new medicines centrally authorised in the EU between 1 January 2010 and 31 December 2014 with at least 1 required paediatric study. For our study cohort of paediatric clinical trials required for these medicines, we used internal EMA databases and publicly available trial registries to determine changes to the planned completion date or study design, rates of trial completion, time to trial completion, and results reporting (peer-reviewed publication or posting on trial registry). Cox proportional hazards regression models were constructed to examine factors associated with study completion. A total of 326 paediatric clinical trials were required for 122 novel medicines authorised by the EMA between 2010 and 2014. In all, 76% (247/326) of paediatric studies were not planned to be completed until after the initial marketing authorisation. The planned completion dates for 50% (162/326) were further postponed by a median of 2.2 years. Overall, 38% (124/326) of paediatric studies were completed as of 30 November 2017. The rate of trial completion for paediatric studies planned to be completed after initial marketing authorisation was 23% (56/247), versus 86% (68/79) for trials planned to be completed before authorisation (adjusted hazard ratio 0.11; 95% CI 0.06–0.19). Among completed studies, the results were reported in a public registry or in the peer-reviewed literature for 85% (105/124) at a median of 1.1 years after study completion, and 60% (74/124) were published in a peer-reviewed journal. Limitations of this study include the potential lack of generalisability to medicines not authorised by the EMA and the possibility for more of these trials to be completed or published in the future.

**Conclusions:**

The completion of many paediatric studies required under the Paediatric Regulation has been delayed. Paediatric studies planned to be completed after marketing authorisation were associated with a lower likelihood of eventual completion, highlighting the need to examine the implementation of current policies in ensuring timely availability of important paediatric information.

## Abstract 2

Since 2007, the European Union has mandated that newly authorized medicines for adults are tested in pediatric trials. In this study, Florence Bourgeois and colleagues evaluate the success of this regulation in terms of planned, completed and reported pediatric trials.

## Author summary

**Why was this study done?:**

- Most new medicines are developed and tested in adults, and clinicians often need to treat paediatric patients with products lacking paediatric safety, efficacy, or dosing information.
- To increase the number of medicines that are appropriately studied in children, the European Union adopted the Paediatric Regulation in 2007, requiring pharmaceutical companies to study new medicines in children.
- Ten years since its implementation, there has been limited assessment of the availability of paediatric trial information resulting from studies required under the regulation.

**What did the researchers do and find?:**

- For all new medicines centrally authorised in the EU between 2010 and 2014, we identified those with paediatric trial requirements under the Paediatric Regulation. A total of 326 paediatric clinical trials were required for 122 medicines and comprised our study cohort.
- After a median follow-up of roughly 7 years, 38% of paediatric trials had been completed, and 17% of medicines had all paediatric requirements fulfilled.
- Most paediatric studies (76%) were not planned to be completed until after marketing authorisation. In addition, delays occurred due to changes in the planned completion date, with 50% of studies extending the completion date at the request of pharmaceutical companies.
- Overall, trials planned to be completed after marketing authorisation were associated with an 89% lower likelihood of completion compared to trials with planned completion before marketing authorisation.
- The results for 85% of completed studies were published or publicly reported in a trial registry, at a median of 1.1 years after the completion date.

**What do these findings mean?:**

- Many paediatric studies required under the Paediatric Regulation have not been completed due to delays.
- Among paediatric trials that were completed, trial results were disseminated in a timely fashion for a majority of the studies.
- Our findings highlight the need to examine the implementation of current policies—including requirements around the timing of trial completion—to ensure timely availability of important paediatric information for new medicines.

## Introduction

Historically, information on the safety, efficacy, and appropriate use of new medicines in children has been lacking [1]. In one study, over 90% of surveyed paediatricians reported prescribing medicines off-label [2]. Over the years, this gap in available evidence has led to serious unintended harms: for example, the off-label paediatric use of paroxetine was associated with an increased risk of suicidal ideation and hostility, resulting in warnings by regulators that the medicine should not be used in children and adolescents [3].

In 2007, the Paediatric Regulation made paediatric development of medicines mandatory in the EU, establishing a system of obligations, rewards, and incentives to promote the study of medicines in children and to improve the information available to clinicians on the use of these products in paediatric populations [4,5]. The requirements were intended to address the longstanding practice in which drugs were developed primarily for adults, and rarely evaluated in children, but were necessarily subsequently used to treat paediatric patients. Under the Paediatric Regulation, before applying for marketing authorisation, pharmaceutical companies must agree with the European Medicines Agency (EMA) Paediatric Committee on a paediatric investigation plan (PIP) aimed at generating the data necessary to authorise the medicine for children. A PIP, which describes the studies to be carried out in children and the timing planned for their completion, is required for all new medicines as well as new indications, new routes of administration, and new pharmaceutical forms of existing authorised products, unless a waiver is granted. A waiver may be granted, for example, because the condition does not occur in children, because the product is unlikely to be effective or safe in children, or because it would not provide a significant benefit compared to products already authorised. The pharmaceutical company can receive a financial reward (a 6-month extension of patent protection for the medicinal product) once it has completed all studies agreed on in the PIP (see Box) [6]. The Paediatric Regulation further provides that the results of paediatric clinical trials must be posted to the EU Clinical Trials Register within 6 months of completion [4].

**Box 1. Key provisions of the EU Paediatric Regulation**

**Entry into force (year):** 2007

**Paediatric development:** Mandatory (unless waived)*

**Main reward:** 6-month extension of the SPC (patent) for non-orphan products or 2-year extension of market exclusivity for orphan products

**Types of products:** New medicinal products; authorised products under patent/SPC if applying for new indication, route, or form

**Excluded products:** Generic, hybrid, well-established use, traditional herbal, homeopathic, and biosimilar products

**Scope of paediatric development:** Derived from adult indication, within same condition

**Timing of PIP or waiver submission:** Early in drug development

**Modification procedure:** Sponsors may propose changes or request a waiver if key elements of an already agreed PIP are no longer workable or appropriate

**Public access to PIP decisions:** Yes

**Require trial registration on public database:** Yes

**Timing of results submission and posting:** Within 6 months of completion

*Full or partial waivers in paediatric subgroups may be granted if the specific medicinal product or a class of medicinal products is likely to be ineffective or unsafe in paediatric patients, if the condition for which the medicine is intended occurs only in adults (or only in some paediatric subsets), or if the medicine does not represent a significant therapeutic benefit over existing treatments for paediatric patients.

EMA, European Medicines Agency; PIP, paediatric investigation plan; SPC, supplementary protection certificate.

The impact of the Paediatric Regulation on the timely completion of and dissemination of results from paediatric trials is uncertain. In principle, the Paediatric Regulation calls for studies to be conducted before marketing authorisation applications are submitted (the Paediatric Regulation states ‘paediatric investigation plans should be submitted early during product development, in time for studies to be conducted in the paediatric population, where appropriate, before marketing authorisation applications are submitted’). However, several factors may cause delays in study completion. For example, a pharmaceutical company can request to complete a study after initial marketing authorisation, allowing the company to apply for marketing authorisation in other age groups (e.g., adults) before starting or completing paediatric studies. The date of completion of these studies can be further postponed at the request of the company via a modification of the PIP as agreed on with the EMA. In addition, the availability of paediatric information may be delayed by varying rates of results reporting after trial completion [7] and completion of clinical trials in the post-authorisation period in general [8–13]. For example, one recent study found that 54% of US Food and Drug Administration (FDA) postmarketing requirements and commitments had been completed after 5 to 6 years of follow-up [9].

In this study, we examined the frequency and duration of delays and the completion of paediatric clinical trials for new medicines centrally authorised in the EU after the entry into force of the Paediatric Regulation. We evaluated the dissemination of the results of these trials through reporting of key trial outcomes in public registries and peer-reviewed publications.

## Methods

### Study cohort

**Fig 1:**
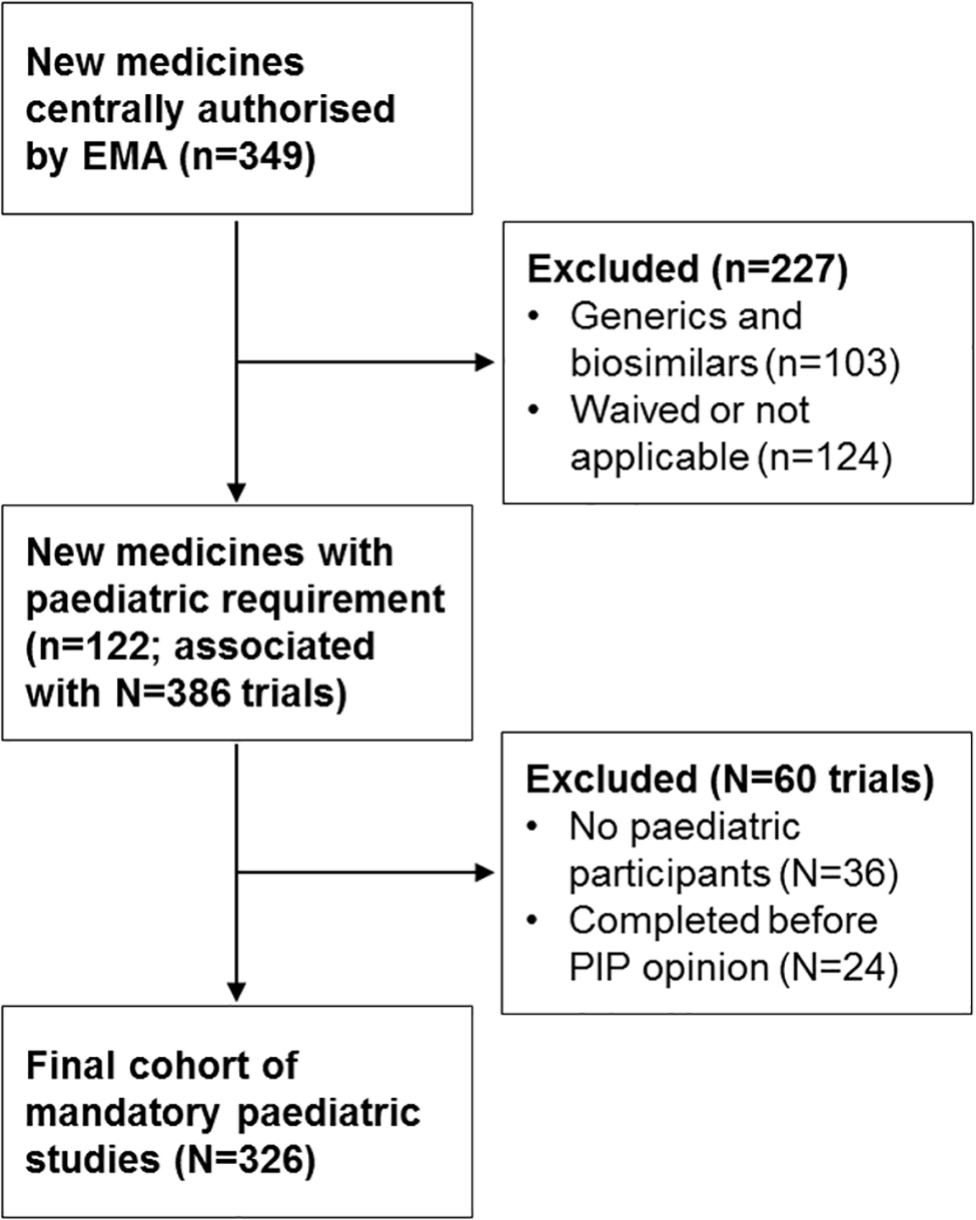
Study flowchart. Lowercase *n*’s refer to the number of medicines; uppercase *N*’s refer to the number of associated paediatric trials identified from the published paediatric investigation plans (PIPs). Traditional herbal and homeopathic products, those authorised under well-established use, and nationally authorised medicines were also excluded.

We used the EMA’s public registry of new marketing authorisations to identify all new medicines centrally authorised in the EU between 1 January 2010 and 31 December 2014 (Fig 1) [14]. The study time frame was chosen to allow at least 2 years of follow-up from the date of marketing authorisation. We included all new medicinal products that were not previously authorised in the EU, including chemical and biologic products and vaccines. Products that were not subject to the obligations of the Paediatric Regulation (e.g., generic drugs; see Box) were excluded from this analysis.

For each identified medicine, we searched the EMA’s public database of opinions and decisions on PIPs to determine whether a PIP had been adopted (leading to publication of a PIP opinion) or if a waiver of paediatric development requirements had been granted for the proposed medical condition [15]. We validated this search with a review of the EMA European public assessment reports, to arrive at a final list of new medicines authorised by the EMA during our study period with paediatric study requirements (S1 Data). Our study cohort was defined as paediatric clinical trials required for these medicines.

### Data extraction

At the medicine level, we first extracted key information on each included medicine, including date of initial marketing authorisation in the EU, indication, therapeutic area (WHO Anatomical Therapeutic Chemical [ATC] code), and orphan status (a designation for medicines authorised for rare diseases).

From the PIP opinion and, if applicable, any subsequent modifications, we then identified all non-duplicate clinical trials included in the PIP and extracted information on the planned start and end dates, size (number of enrolled participants), study population, and study type. We categorised study type based on the primary study endpoints as (i) primarily efficacy studies, (ii) efficacy and safety studies, (iii) primarily safety studies, or (iv) pharmacokinetic/pharmacodynamics (PK/PD) and dose-finding studies (hereafter ‘PK/PD studies’). We excluded studies included in PIPs that did not plan to enrol any paediatric participants (e.g., adult PK/PD or bioequivalence studies; *N* = 36) and trials whose completion dates predated the date when the PIP opinion was published (*N* = 24). Data extraction was conducted by 2 investigators (TJH, FTB), with conflicts resolved by consensus.

For each paediatric study, we tracked changes in the study design and timeline. Once a PIP is published, changes to paediatric studies can only be made through 1 or more procedures of modification of the agreed PIP. These modification procedures can only be initiated at the request of the sponsor. Using EMA’s internal database of paediatric applications, we identified changes in the expected size of the trial, study endpoints, study populations, statistical methods, or treatment duration, and any other substantive changes to the trial. We also identified changes in the planned completion date. The number and types of changes were extracted for all modification procedures that had been completed and published by the EMA’s Paediatric Committee through 31 January 2017.

To determine whether a study had been completed, we searched the EU Clinical Trials Register (https://www.clinicaltrialsregister.eu) and the US National Institutes of Health’s ClinicalTrials.gov registry (https://clinicaltrials.gov/), using the study name or a combination of the medicine name, study population, study size, planned completion date, and study type. Both the US and EU have requirements on trial registration and results posting that should technically cover all included paediatric trials. In the EU, since 2007, paediatric studies are required to be registered in the EU Clinical Trials Register, and the results posted within 6 months of study completion. In the US, applicable clinical trials initiated after 27 September 2007 are required to be registered in ClinicalTrials.gov and to post results within 1 year after completion of data collection for the primary outcome. We crosschecked the trial status reported on public registries with compliance checks conducted by the EMA on PIP studies [16] and by searching for other public and commercial mentions of the trial and its completion status.

Finally, we defined results reporting as a composite endpoint of (i) results posted on the EU Clinical Trials Register or ClinicalTrials.gov and/or (ii) publication in a peer-reviewed journal. As described above, we searched for paediatric trials on the EU Clinical Trials Register and ClinicalTrials.gov and then identified whether and when results had been posted. We searched Medline, Embase, and Web of Science for peer-reviewed publications of the paediatric trial, and, for each publication, extracted the date of first publication online (if earlier than the publication date listed in the citation).

### Statistical analyses

We assessed the time difference between the initially planned paediatric trial completion date and the date of initial marketing authorisation granted by the European Commission (representing when the medicine is first available on the market) as well as the duration of granted extensions (i.e., delays) to the planned completion date. We calculated the unadjusted rate and relative risk of study completion, the rate of completion of all clinical studies specified in a given PIP, and the rate and median time to results reporting.

In adjusted analyses, we constructed multivariable logistic regression models to examine factors associated with the likelihood of delays and Cox proportional hazards regression models to examine factors associated with study completion. Models included all variables of interest regardless of statistical significance: therapeutic area (ATC code), study type, an indicator variable for trials planned to be completed after (versus before) marketing authorisation, and a time variable for the PIP opinion. In all models, robust standard errors were clustered by PIP.

All study analyses described above were conducted according to the project protocol agreed upon prior to data collection, with a database lock in November 2017 (S1 Protocol). In a pre-specified sensitivity analysis, we repeated our analysis of trial completion and results reporting excluding PK/PD studies in order to address the possibility that PK/PD studies may be easier to complete (and therefore more likely to be completed) than other studies. Since the conduct of required paediatric studies may differ for medicines with versus without any initially intended paediatric populations, we also performed a post hoc sensitivity analysis limiting our analysis to the cohort of medicines authorised only in adults.

Statistical analyses were performed using Stata version 12 (StataCorp), and 2-tailed *P* values < 0.05 were considered statistically significant.

## Results

Of 246 new medicines (excluding generic and biosimilar products) that were authorised by the EMA during our study period, 122 (50%) with PIPs were included (Fig 1); most of these medicines were anti-infective (34, 28%), antineoplastic or immunomodulatory (23, 19%), or alimentary or metabolic agents (17, 14%). These 122 medicines with PIPs were associated with 326 paediatric clinical trials, cumulatively planning to enrol 51,324 participants (Table 1; Fig 1). In all, 182 of 326 trials (56%) were primarily efficacy studies, 21 (6%) trials had both primary efficacy and safety endpoints, 70 (21%) trials had only safety primary endpoints, and 53 (16%) trials were PK/PD studies. The median duration of follow-up for all studies from the date of PIP publication to 30 November 2017 was 7.6 years (IQR 6.5–8.4 years).

**Table 1:** Characteristics of paediatric trials (*N* = 326) for new medicines authorised by the EMA in 2010–2014.

Characteristic	Number (%)
Therapeutic area	
Alimentary and metabolism	44 (13.5%)
Blood	43 (13.2%)
Cardiovascular	14 (4.3%)
Genitourinary	9 (2.8%)
Anti-infective	75 (23.0%)
Antineoplastic and immunomodulatory	44 (13.5%)
Neurologic	53 (16.3%)
Respiratory	28 (8.6%)
Musculoskeletal and others	16 (4.9%)
PIP opinion year	
2008	40 (12.3%)
2009	110 (33.7%)
2010	62 (19.0%)
2011	64 (19.6%)
2012	43 (13.2%)
2013	7 (2.2%)
Study type	
PK/PD	53 (16.3%)
Primarily efficacy	182 (55.8%)
Efficacy and safety	21 (6.4%)
Primarily safety	70 (21.5%)
Planned trial completion before or after marketing authorisation	
After	247 (75.8%)
Before	79 (24.2%)
Any extension of completion date	
Yes	162 (49.7%)
No	164 (50.3%)
Any modification (excluding extensions)	
Yes	201 (61.7%)
No	125 (38.3%)
Orphan drug status	
Yes	48 (14.7%)
No	278 (85.3%)

The study cohort of paediatric trials relates to 122 new medicines that were centrally authorised by the EMA between 2010 and 2014 with paediatric requirements, of which 86 were initially authorised for use in adults only (see Methods for details on cohort construction and definitions).

EMA, European Medicines Agency; PIP, paediatric investigation plan; PK/PD, pharmacokinetic/pharmacodynamics.

### Delays and modifications of paediatric clinical trials

Most (247, 76%) paediatric studies were not planned to be completed until after the initial marketing authorisation. Overall, the originally planned completion dates for 162 (50%) paediatric studies (representing 78 [64%] of medicines) were extended through modification of the PIP at the request of companies, resulting in a median delay of 2.2 years (IQR 1.1–3.8 years).

The majority (201, 62%) of paediatric studies had at least 1 modification (other than changes to planned study completion date) after publication of the PIP, with a median of 2 modifications. Among these, 85 (26%) trials had changes to study population or inclusion/exclusion criteria, 83 (25%) trials had changes to sample size, 73 (22%) had changes to statistical methods, and 69 (21%) had changes to study endpoints.

Most (60 of 83, 72%) modifications to sample size resulted in trials that were smaller than originally planned. Among these trials with sample size modification, the median reduction in trial size was 29% (IQR 20%–50%), corresponding to 4,032 total fewer participants and a median reduction of 20 participants (IQR 10–77). The total number of modifications was significantly associated with delays in both univariable (*P* = 0.001) and multivariable logistic regression analyses (adjusted odds ratio 2.31; 95% CI 1.18–4.51; *P* = 0.02) (S1 Table).

### Completion of paediatric clinical trials

As of 30 November 2017, 124 of 326 (38%) paediatric clinical trials were completed, and all clinical studies specified in the PIP were completed for 21 of 122 (17%) medicines. The rates of trial completion 1, 2, and 5 years following PIP publication were 4%, 14%, and 34%, respectively. The rates of completion were 51% for PK/PD studies, 32% for primarily efficacy studies, 38% for trials with both primary efficacy and safety endpoints, and 43% for primarily safety studies (Table 2). There were no statistically significant differences in time to completion by study type. For completed trials, the median time to completion from the publication of the PIP opinion was 3 years (IQR 1.5 to 4.4 years), and the median difference in planned and reported completion dates was 0.2 years (IQR −0.3 to 1.3 years).

**Table 2:** Unadjusted rates of study completion and results reporting.

Characteristic	Study completion, *N* (%)	*P* value	Results reporting, *N* (%)	*P* value
Therapeutic area				
Alimentary and metabolism	16 (36.4%)	<0.001	13 (81.3%)	0.06
Blood	21 (48.8%)		16 (76.2%)	
Cardiovascular	3 (21.4%)		3 (100.0%)	
Genitourinary	2 (22.2%)		1 (50.0%)	
Anti-infective	35 (46.7%)		29 (82.9%)	
Antineoplastic and immunomodulatory	6 (13.6%)		3 (50.0%)	
Neurologic	17 (32.1%)		16 (94.1%)	
Respiratory	22 (78.6%)		22 (100.0%)	
Musculoskeletal and others	2 (12.5%)		2 (100.0%)	
Planned trial completion before or after marketing authorization				
After	56 (22.7%)	<0.001	47 (83.9%)	0.51
Before	68 (86.1%)		58 (85.3%)	
Orphan drug status				
Yes	18 (37.5%)	0.56	18 (100.0%)	0.04
No	106 (38.1%)		87 (82.1%)	
Study type				
PK/PD	27 (50.9%)	0.08	18 (66.7%)	0.04
Primarily efficacy	59 (32.4%)		52 (88.1%)	
Efficacy and safety	8 (38.1%)		7 (87.5%)	
Primarily safety	30 (42.9%)		28 (93.3%)	
Any extension granted				
Yes	59 (36.4%)	0.28	49 (83.1%)	0.41
No	65 (39.6%)		56 (86.2%)	
Any modification granted				
Yes	90 (44.8%)	0.01	73 (81.1%)	0.06
No	34 (27.2%)		32 (94.1%)	

The composite endpoint of results reporting refers to (i) results posted on the EU Clinical Trials Register or ClinicalTrials.gov and/or (ii) publication in a peer-reviewed journal. Results reporting is determined for completed trials only.

PK/PD, pharmacokinetic/pharmacodynamics.

**Fig 2:**
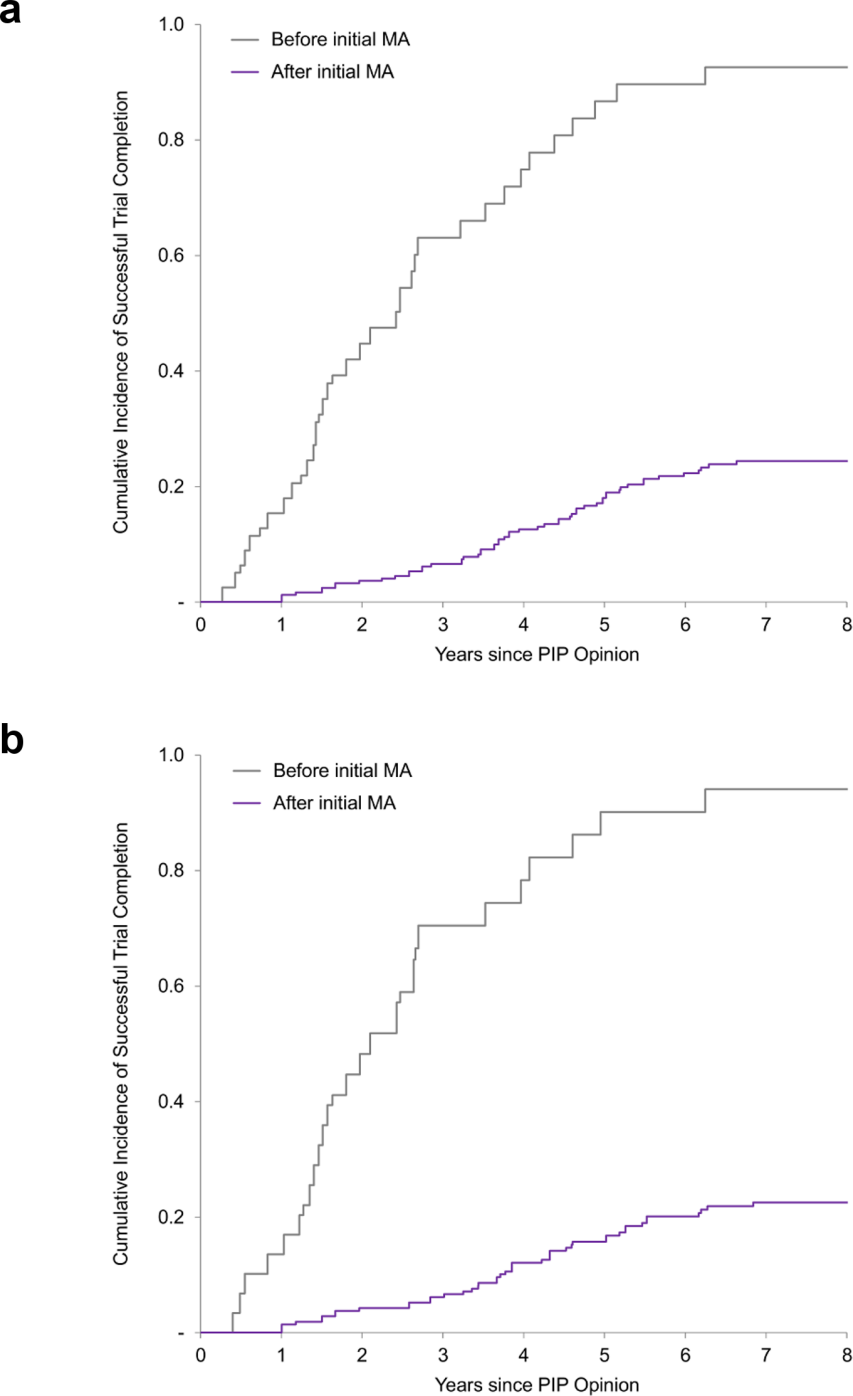
Cumulative incidence curves of paediatric trial completion. (a) Cumulative incidence rates of trial completion for all paediatric trials. (b) Cumulative incidence rates of trial completion for efficacy and safety trials (i.e., excluding PK/PD studies). Before/after initial MA refers to the planned completion date relative to the actual initial MA date. MA, marketing authorisation; PIP, paediatric investigation plan; PK/PD, pharmacokinetic/pharmacodynamics.

The unadjusted rate of trial completion for paediatric studies planned to be completed after initial marketing authorisation was 23% (56 of 247), versus 86% (68 of 79) for trials planned to be completed before authorisation (risk ratio 0.26; 95% CI 0.21–0.34; *P <* 0.001). In multivariable Cox regression models, trials planned to be completed after authorisation were associated with an 89% lower likelihood of completion (hazard ratio [HR] 0.11; 95% CI 0.06–0.19; *P <* 0.001) than trials planned to be completed before marketing authorisation (Fig 2). Similar results were obtained in a sensitivity analysis excluding PK/PD studies (S2 Table).

Similar results were also obtained in a sensitivity analysis limiting our analysis to paediatric trials required for the 86 medicines initially authorised only in adults (S3 Table). As of 30 November 2017, 19% (39/203) of clinical trials for medicines authorised only in adults were completed, compared to 38% for the full dataset. Trials planned to be completed after authorisation were associated with a similarly lower likelihood of completion (11% for studies planned to be completed after authorisation versus 79% for studies planned to be completed before authorisation; HR 0.07; 95% CI 0.02–0.23; *P <* 0.001).

### Results reporting and publication of paediatric clinical trials

Among the 124 completed paediatric trials, the results were reported for 105 (85%) (Table 2). The results for 63 (51%) trials were published both in a trial registry and a journal, the results for 31 (25%) trials were posted only in a trial registry, and the results for 11 (9%) trials were published in a journal but not available in a trial registry.

The median time to the composite endpoint of first results reporting (either in a trial registry or peer-reviewed journal) was 4.7 years (IQR 3.2 to 5.8 years) from the date of publication of the PIP, 1.8 years (IQR −0.1 to 2.8 years) from the date of initial marketing authorisation, and 1.1 years (IQR 0.7 to 1.6 years) from the trial completion date. Substantively similar results were obtained with logistic regression models.

## Discussion

In this study of mandatory paediatric study requirements under the EU’s Paediatric Regulation, we found that most paediatric studies have not yet been completed for new medicines that were authorised for adult use between 2010 and 2014. After a median follow-up of 7 years from publication of the PIP, 17% of medicines initially authorised for adults had completed all required paediatric clinical trials, and 38% of paediatric studies had been completed. In addition, paediatric studies that were planned to be completed after initial marketing authorisation were 89% less likely to be completed than studies planned to be completed before authorisation. This difference in likelihood of completion remained significant when considering only efficacy and safety trials. Once trials were completed, the results for 85% of completed studies were publicly reported in a trial registry or a peer-reviewed journal at a median of 1.1 years after completion.

To our knowledge, this is the first study of the completion and results reporting of trials required under the Paediatric Regulation, providing evidence on the performance of this regulation in achieving important public health goals. This is timely as 2017 marks the 10-year anniversary of the Paediatric Regulation’s entry into force in the EU [17,18]. The experience over the past decade indicates that the Paediatric Regulation has contributed to increasing numbers of paediatric trials and medicines available to children [18]. Yet, the full potential of the Paediatric Regulation may not be realised if required paediatric studies are not completed and reported rapidly and adequately. It is possible that delays in the conduct of paediatric trials are justifiable in some cases, for instance if negative safety signals are observed in adult trials. However, extensive delays in study completion hinder the ability of regulators and stakeholders to achieve one of the primary aims of the Paediatric Regulation: to reduce the rate of off-label use. The European Commission notes that deferred studies may become further delayed because of recruitment difficulties once the product is available on the market, since parents may be less willing to have their child participate in a trial if the medicine can already be prescribed (even if off-label) [19].

Our study indicates that paediatric studies that are planned to be completed after marketing authorisation are indeed less likely to be completed in a timely manner. These findings may also help inform the oversight of paediatric studies in the US. In contrast to the EU’s Paediatric Regulation, which is both mandatory and provides for a reward, in the US, the FDA operates 2 separate programmes to encourage paediatric drug development: one (the Best Pharmaceuticals for Children Act) provides incentives but is voluntary [20,21], while the other (the Pediatric Research Equity Act) establishes requirements for conducting certain paediatric studies but does not provide incentives for doing so. Although the underlying legal bases differ between the US and the EU, our study findings are broadly consistent with the experience with paediatric studies in the US. Most paediatric studies that are required by the FDA are similarly deferred [22,23], and, as observed in this study, extension requests are frequently granted. As of December 2016, the FDA reports that 71% (262/369) of deferred studies were granted further extensions of their completion dates [24]. Finally, a recent study of drugs approved by the FDA from 2003 to 2012 reported that 35% of drugs approved by the FDA without paediatric assessments eventually submitted paediatric data, with a mean follow-up of 8.6 years from approval [25].

Our findings underscore the lack of effective tools to enforce completion of important paediatric studies once medicines become available on the market. Under the Paediatric Regulation, the European Commission may impose financial penalties for infringement of the obligations of the regulation, although no penalties have been assessed to date [4]. One approach to improve the completion of paediatric studies could be to require more studies to be completed before validating the initial marketing authorisation submission [18]. Another potential approach could be to increase the reward, or to be able to tailor it using a pre-specified sliding scale, depending on the time it takes for required studies to be completed. Finally, the conditions for allowing extensions to the planned completion date could be reviewed to ensure that such requests account for the potential harms caused by delays in access to information.

There were a number of limitations to our analysis. First, although our study provided a median of 7 years of follow-up from the first publication date of the paediatric study requirements for the included medicines, it is possible that more of the required trials for these medicines may be completed or published in the future. It is uncertain if and when such trials will actually be completed. In one large study of paediatric clinical drug trials, the median trial duration was 1.8 years [26]. Second, despite legal requirements for the registration and public reporting of trial status, we cannot exclude the possibility that an additional number of apparently incomplete trials may have already been completed. However, we crosschecked our results across public and EMA internal regulatory sources, and we do not believe this possibility would substantially affect our study conclusions. We note that for all trials that were subject to compliance checks by the EMA, the trial status reported to the EMA was consistent with that reported on public registries. Finally, our study included paediatric studies required for new medicines centrally authorised by the EMA, and our findings may not apply to unapproved products or to the relatively limited number of medicines that are authorised at the national level.

## Conclusions

The Paediatric Regulation was introduced in response to the serious harms caused by exposing children to unauthorised and inadequately tested medicines. Our findings suggest that many required paediatric studies have not been completed, with studies that are postponed to after authorisation significantly less likely to be completed than those performed before authorisation. Policies to limit delays in study completion could accelerate the availability of clinically valuable information on new medicines for children.

## Supporting information

S1 Data Medicines authorised by the EMA in the study period with paediatric investigation plans (PIPs). (PDF)

S1 Protocol (PDF)

S1 Table Results from multivariable logistic regression models of study delay. Results from multivariable logistic regression models for odds of delay to study completion (i.e., extension granted to planned completion date). Antineoplastic (ATC code L) also includes immunomodulatory agents. ^a^World Health Organization ATC therapeutic area. ^b^Omitted due to collinearity. MA, marketing authorisation.
(DOCX)

S2 Table Results from multivariable Cox regression models of trial completion. Hazard ratios (HRs) and CIs are from multivariable Cox regression models for trial completion of all trials (i.e., including trials required for medicines authorised for use in any paediatric age group) and of efficacy and safety trials only (efficacy/safety; i.e., excluding PK/PD studies). Antineoplastic (ATC code L) also includes immunomodulatory agents. ^a^World Health Organization ATC therapeutic area. ^b^Omitted due to collinearity. MA, marketing authorisation; PK/PD, pharmacokinetic/pharmacodynamics.
(DOCX)

S3 Table Characteristics of paediatric trials for new medicines initially authorised for adults only in 2010–2014. The study cohort of paediatric trials relates to 122 new medicines that were centrally authorised by the EMA between 2010 and 2014 with paediatric requirements, of which 86 were initially authorised for use in adults only (see Methods for details on cohort construction and definitions). PIP, paediatric investigation plan; PK/PD, pharmacokinetic/pharmacodynamics.
(DOCX)

## Abbreviations

ATC: Anatomical Therapeutic Chemical
EMA: European Medicines Agency
FDA: US Food and Drug Administration
HR: hazard ratio
PIP: paediatric investigation plan
PK/PD: pharmacokinetic/pharmacodynamics
