# Supplementary material for: Delays in completion and results reporting of clinical trials under the Paediatric Regulation in the European Union: A cohort study
Source: PLoS Med. 2018 Mar 1;15(3):e1002520. doi: 10.1371/journal.pmed.1002520 (PMC5832187; doi:10.1371/journal.pmed.1002520)
Supplement: S1 Data — (PDF) [file pmed.1002520.s001.pdf]

S1 Data. Medicines authorised by EMA in study period with paediatric investigation plans (PIP)

Medicine name	Common name	Authorisation date	Indication	ATC code (primary)	Conditional	Exceptional	Orphan
Rixubis	nonacog gamma	12/19/2014	Treatment and prophylaxis of bleeding in patients with haemophilia B (congenital factor IX deficiency).	B	no	no	no
Moventig	naloxegol oxalate	12/8/2014	Treatment of opioid-induced constipation (OIC) in adult patients who have had an inadequate response to laxative(s).	A	no	no	no
Trulicity	dulaglutide	11/21/2014	Trulicity is indicated in adults with type-2 diabetes mellitus to improve glycaemic control as: MonotherapyWhen diet and exercise alone do not provide adequate glycaemic control in patients for whom the use of metformin is considered inappropriate due to intolerance or contraindications. Add-on therapyIn combination with other glucose-lowering medicinal products including insulin, when these, together with diet and exercise, do not provide adequate glycaemic control.	A	no	no	no
Lymphoseek	tilmanocept	11/19/2014	This medicinal product is for diagnostic use only. Radiolabelled Lymphoseek is indicated for imaging and intraoperative detection of sentinel lymph nodes draining a primary tumour in adult patients with breast cancer, melanoma, or localised squamous cell carcinoma of the oral cavity. External imaging and intraoperative evaluation may be performed using a gamma detection device.	V	no	no	no
Rezolsta	darunavir / cobicistat	11/19/2014	Rezolsta, is indicated in combination with other antiretroviral medicinal products for the treatment of human immunodeficiency virus 1 (HIV 1) infection in adults aged 18 years or	J	no	no	no

Medicine name	Common name	Authorisation date	Indication	ATC code (primary)	Conditional	Exceptional	Orphan
			older.<br>Genotypic testing should guide the use of Rezolsta.				
Zydelig	idelalisib	9/18/2014	Zydelig is indicated in combination with an antiCD20 monoclonal antibody (rituximab or ofatumumab) for the treatment of adult patients with chronic lymphocytic leukaemia (CLL):<br><br>who have received at least one prior therapy (see section4.4), or<br>as first line treatment in the presence of 17pdeletion or TP53mutation in patients who are not eligible for any other therapies (see section4.4).<br><br>Zydelig is indicated as monotherapy for the treatment of adult patients with follicular lymphoma (FL) that is refractory to two prior lines of treatment (see section4.4).	L	no	no	no
Triumeq	abacavir sulfate / dolutegravir sodium / lamivudine	9/1/2014	Triumeq is indicated for the treatment of Human Immunodeficiency Virus (HIV) infected adults and adolescents above 12 years of age weighing at least 40 kg.<br>Before initiating treatment with abacavir-containing products, screening for carriage of the HLA-B*5701 allele should be performed in any HIV-infected patient, irrespective of racial origin. Abacavir should not be used in patients known to carry the HLA-B*5701 allele.	J	no	no	no
Daklinza	daclatasvir dihydrochloride	8/22/2014	Daklinza is indicated in combination with other medicinal products for the treatment of chronic hepatitis C virus (HCV) infection in adults (see sections 4.2, 4.4 and 5.1).<br>For HCV genotype specific activity, see sections 4.4 and 5.1.	J	no	no	no

Medicine name	Common name	Authorisation date	Indication	ATC code (primary)	Conditional	Exceptional	Orphan
Translarna	ataluren	7/31/2014	Translarna is indicated for the treatment of Duchenne muscular dystrophy resulting from a nonsense mutation in the dystrophin gene, in ambulatory patients aged 5 years and older.	N	yes	no	yes
Gazyvaro	obinutuzumab	7/23/2014	Chronic Lymphocytic Leukaemia (CLL)Gazyvaro in combination with chlorambucil is indicated for the treatment of adult patients with previously untreated chronic lymphocytic leukaemia (CLL) and with comorbidities making them unsuitable for full-dose fludarabine based therapy (see section 5.1).Follicular Lymphoma (FL) Gazyvaro in combination with bendamustine followed by Gazyvaro maintenance is indicated for the treatment of patients with follicular lymphoma (FL) who did not respond or who progressed during or up to 6 months after treatment with rituximab or a rituximab-containing regimen.	L	no	no	yes
Nuwiq	simoctocog alfa	7/22/2014	Treatment and prophylaxis of bleeding in patients with haemophilia A (congenital factor VIII deficiency). Nuwiq can be used for all age groups.	B	no	no	no
Plegridy	peginterferon beta-1a	7/18/2014	Treatment of relapsing remitting multiple sclerosis in adult patients.	L	no	no	no
Mekinist	trametinib	6/30/2014	Trametinib as monotherapy or in combination with dabrafenib is indicated for the treatment of adult patients with unresectable or metastatic melanoma with a BRAF V600 mutation.<br>Trametinib monotherapy has not demonstrated clinical activity in patients who have progressed on a prior BRAF inhibitor therapy.	L	no	no	no

Medicine name	Common name	Authorisation date	Indication	ATC code (primary)	Conditional	Exceptional	Orphan
Entyvio	vedolizumab	5/22/2014	Ulcerative colitisEntyvio is indicated for the treatment of adult patients with moderately to severely active ulcerative colitis who have had an inadequate response with, lost response to, or were intolerant to either conventional therapy or a tumour necrosis factor alpha (TNF) antagonist.<br>Crohns disease Entyvio is indicated for the treatment of adult patients with moderately to severely active Crohns disease who have had an inadequate response with, lost response to, or were intolerant to either conventional therapy or a tumour necrosis factor alpha (TNF) antagonist.	L	no	no	no
Jardiance	empagliflozin	5/22/2014	Jardiance is indicated in the treatment of type-2 diabetes mellitus to improve glycaemic control in adults as:MonotherapyWhen diet and exercise alone do not provide adequate glycaemic control in patients for whom use of metformin is considered inappropriate due to intolerance.Add-on combination therapyIn combination with other glucoselowering medicinal products including insulin, when these, together with diet and exercise, do not provide adequate glycaemic control.	A	no	no	no
Olysio	simeprevir	5/14/2014	Olysio is indicated in combination with other medicinal products for the treatment of chronic hepatitis C (CHC) in adult patients.<br>For hepatitis C virus (HCV) genotype specific activity, see sections 4.4 and 5.1.	J	no	no	no
Adempas	riociguat	3/27/2014	Chronic thromboembolic pulmonary hypertension (CTEPH) Adempas is indicated for the treatment of adult	C	no	no	yes

Medicine name	Common name	Authorisation date	Indication	ATC code (primary)	Conditional	Exceptional	Orphan
			patients with WHO Functional Class (FC) II to III with<br><br>inoperable CTEPH,<br>persistent or recurrent CTEPH after surgical treatment,<br><br>to improve exercise capacity (see section 5.1). Pulmonary arterial hypertension (PAH) Adempas, as monotherapy or in combination with endothelin receptor antagonists, is indicated for the treatment of adult patients with pulmonary arterial hypertension (PAH) with WHO Functional Class (FC) II to III to improve exercise capacity. Efficacy has been shown in a PAH population including aetiologies of idiopathic or heritable PAH or PAH associated with connective tissue disease (see section 5.1).				
Cometriq	cabozantinib	3/21/2014	Treatment of adult patients with progressive, unresectable locally advanced or metastatic medullary thyroid carcinoma.	L	yes	no	yes
Eperzan	albiglutide	3/21/2014	Eperzan is indicated for the treatment of type 2 diabetes mellitus in adults to improve glycaemic control as:<br>Monotherapy<br>When diet and exercise alone do not provide adequate glycaemic control in patients for whom use of metformin is considered inappropriate due to contraindications or intolerance.<br>Add-on combination therapy<br>In combination with other glucose-lowering medicinal products including basal insulin, when these, together with diet and exercise, do not provide adequate glycaemic	A	no	no	no

Medicine name	Common name	Authorisation date	Indication	ATC code (primary)	Conditional	Exceptional	Orphan
			control (see section 4.4 and 5.1 for available data on different combinations).				
Latuda	lurasidone	3/21/2014	Treatment of schizophrenia in adults aged 18 years and over.	N	no	no	no
Izba	travoprost	2/20/2014	Decrease of elevated intraocular pressure in adult patients with ocular hypertension or open-angle glaucoma	S	no	no	no
Tecfidera	dimethyl fumarate	1/30/2014	Treatment of adult patients with relapsing remitting multiple sclerosis.	N	no	no	no
Sovaldi	sofosbuvir	1/16/2014	Sovaldi is indicated in combination with other medicinal products for the treatment of chronic hepatitis C (CHC) in adults.	J	no	no	no
Tivicay	dolutegravir	1/16/2014	Tivicay is indicated in combination with other anti-retroviral medicinal products for the treatment of Human Immunodeficiency Virus (HIV) infected adults and adolescents above 12 years of age.	J	no	no	no
Opsumit	macitentan	12/20/2013	Opsumit, as monotherapy or in combination, is indicated for the long-term treatment of pulmonary arterial hypertension (PAH) in adult patients of WHO Functional Class (FC) II to III.<br>Efficacy has been shown in a PAH population including idiopathic and heritable PAH, PAH associated with connective tissue disorders, and PAH associated with corrected simple congenital heart disease.	C	no	no	yes
Brintellix	vortioxetine	12/18/2013	Treatment of major depressive episodes in adults	N	no	no	no
Abilify Maintena	aripiprazole	11/15/2013	Maintenance treatment of schizophrenia in adult patients stabilised with oral aripiprazole.	N	no	no	no
Invokana	canagliflozin	11/15/2013	Invokana is indicated in adults aged 18 years and older with type 2 diabetes mellitus to improve glycaemic control as:<br>Monotherapy	A	no	no	no

Medicine name	Common name	Authorisation date	Indication	ATC code (primary)	Conditional	Exceptional	Orphan
			When diet and exercise alone do not provide adequate glycaemic control in patients for whom the use of metformin is considered inappropriate due to intolerance or contraindications.<br>Add on therapy<br>Add on therapy with other glucose-lowering medicinal products including insulin, when these, together with diet and exercise, do not provide adequate glycaemic control (see sections 4.4, 4.5, and 5.1 for available data on different add on therapies).				
Relvar Ellipta	fluticasone furoate / vilanterol	11/13/2013	Asthma Indication:<br>Relvar Ellipta is indicated in the regular treatment of asthma in adults and adolescents aged 12 years and older, where use of a combination product (long-acting beta2-agonist and inhaled corticosteroid) is appropriate:<br><br>patients not adequately controlled with inhaled corticosteroids and as needed inhaled short acting beta2-agonists.<br><br>COPD Indication:<br>Relvar Ellipta is indicated for the symptomatic treatment of adults with COPD with a FEV1 70% predicted normal (post-bronchodilator) in patients with an exacerbation history despite bronchodilator therapy.	R	no	no	no
NovoEight	turoctocog alfa	11/13/2013	Treatment and prophylaxis of bleeding in patients with haemophilia A (congenital factor VIII deficiency). Novoeight can be used for all age groups.	B	no	no	no
Evarrest	human fibrinogen / human thrombin	9/25/2013	Evarrest is indicated in adults for supportive treatment in surgery	B	no	no	no

Medicine name	Common name	Authorisation date	Indication	ATC code (primary)	Conditional	Exceptional	Orphan
			where standard surgical techniques are insufficient, for improvement of haemostasis.				
Vipidia	alogliptin	9/19/2013	Vipidia is indicated in adults aged 18 years and older with type 2 diabetes mellitus to improve glycaemic control in combination with other glucose lowering medicinal products including insulin, when these, together with diet and exercise, do not provide adequate glycaemic control (see sections 4.4, 4.5 and 5.1 for available data on different combinations).	A	no	no	no
Lemtrada	alemtuzumab	9/12/2013	Lemtrada is indicated for adult patients with relapsing-remitting multiple sclerosis (RRMS) with active disease defined by clinical or imaging features.	L	no	no	no
Aubagio	teriflunomide	8/26/2013	Treatment of adult patients with relapsing-remitting multiple sclerosis (MS).	L	no	no	no
Stivarga	regorafenib	8/26/2013	Stivarga is indicated for the treatment of adult patients with:<br><br>metastatic colorectal cancer (CRC) who have been previously treated with, or are not considered candidates for, available therapies - these include fluoropyrimidine-based chemotherapy, an anti-VEGF therapy and an anti-EGFR therapy; unresectable or metastatic gastrointestinal stromal tumors (GIST) who progressed on or are intolerant to prior treatment with imatinib and sunitinib.	L	no	no	no
Tafinlar	dabrafenib	8/26/2013	Dabrafenib as monotherapy or in combination with trametinib is indicated for the treatment of adult patients with unresectable or metastatic melanoma with a BRAF V600 mutation.	L	no	no	no

Medicine name	Common name	Authorisation date	Indication	ATC code (primary)	Conditional	Exceptional	Orphan
			Dabrafenib as monotherapy or in combination with trametinib is indicated for the treatment of adult patients with unresectable or metastatic melanoma with a BRAF V600 mutation.				
Voncento	human coagulation factor VIII / human von willebrand factor	8/12/2013	Von Willebrand disease (VWD) Prophylaxis and treatment of haemorrhage or surgical bleeding in patients with VWD, when desmopressin (DDAVP) treatment alone is ineffective or contraindicated. Haemophilia A (congenital factor-VIII deficiency) Prophylaxis and treatment of bleeding in patients with haemophilia A.	B	no	no	no
Lojuxta	lomitapide	7/31/2013	Lojuxta is indicated as an adjunct to a lowfat diet and other lipidlowering medicinal products with or without low-density-lipoprotein (LDL) apheresis in adult patients with homozygous familial hypercholesterolaemia (HoFH). Genetic confirmation of HoFH should be obtained whenever possible. Other forms of primary hyperlipoproteinaemia and secondary causes of hypercholesterolaemia (e.g. nephrotic syndrome, hypothyroidism) must be excluded.	C	no	yes	no
Lonquex	lipegfilgrastim	7/25/2013	Reduction in the duration of neutropenia and the incidence of febrile neutropenia in adult patients treated with cytotoxic chemotherapy for malignancy (with the exception of chronic myeloid leukaemia and myelodysplastic syndromes).	L	no	no	no
Iclusig	ponatinib	7/1/2013	Iclusig is indicated in adult patients with:	L	no	no	yes

Medicine name	Common name	Authorisation date	Indication	ATC code (primary)	Conditional	Exceptional	Orphan
			chronic-phase, accelerated-phase or blast-phase chronic myeloid leukaemia (CML) who are resistant to dasatinib or nilotinib, who are intolerant to dasatinib or nilotinib and for whom subsequent treatment with imatinib is not clinically appropriate, or who have the T315I mutation; Philadelphia-chromosome-positive acute lymphoblastic leukaemia (Ph+ ALL) who are resistant to dasatinib, who are intolerant to dasatinib and for whom subsequent treatment with imatinib is not clinically appropriate, or who have the T315I mutation.				
Maci	autologous cultured chondrocytes	6/27/2013	Repair of symptomatic cartilage defects of the knee.	M	no	no	no
Bosulif	bosutinib (as monohydrate)	3/27/2013	Bosulif is indicated for the treatment of adult patients with chronic-phase, accelerated-phaseand blast-phasePhiladelphia-chromosome-positive chronic myelogenous leukaemiapreviously treated with one or more tyrosine-kinase inhibitors and for whom imatinib, nilotinib and dasatinib are not considered appropriate treatment options.	L	yes	no	yes
Adasuve	loxapine	2/20/2013	Adasuve is indicated for the rapid control of mild-to-moderate agitation in adult patients with schizophrenia or bipolar disorder. Patients should receive regular treatment immediately after control of acute agitation symptoms.	N	no	no	no
Lyxumia	lixisenatide	2/1/2013	Lyxumia is indicated for the treatment of adults with type 2 diabetes mellitus to achieve glycaemic control in combination with oral glucose lowering medicinal products and/or basal insulin when these, together with diet and exercise, do not provide adequate	A	no	no	no

Medicine name	Common name	Authorisation date	Indication	ATC code (primary)	Conditional	Exceptional	Orphan
			glycaemic control (see sections 4.4 and 5.1 for available data on the different combinations).				
BindRen	colestilan	1/21/2013	Treatment of hyperphosphataemia in adult patients with chronic kidney diseasestage 5 receiving haemodialysis or peritoneal dialysis.	V	no	no	no
Tresiba	insulin degludec	1/21/2013	Treatment of diabetes mellitus in adults.	A	no	no	no
Ryzodeg	insulin aspart / insulin degludec	1/21/2013	Treatment of diabetes mellitus in adults, adolescents and children from the age of 2 years.	A	no	no	no
Betmiga	mirabegron	12/20/2012	Symptomatic treatment of urgency. Increased micturition frequency and / or urgency incontinence as may occur in adult patients with overactive-bladdersyndrome.	G	no	no	no
NexoBrid	concentrate of proteolytic enzymes enriched in bromelain	12/18/2012	NexoBrid is indicated for removal of eschar in adults with deep partial- and full-thickness thermal burns.	D	no	no	yes
Constella	linaclotide	11/26/2012	Constella is indicated for the symptomatic treatment of moderate to severe irritable-bowel syndrome with constipation (IBS-C) in adults.	A	no	no	no
Glybera	alipogene tiparvovec	10/25/2012	Glybera is indicated for adult patients diagnosed with familial lipoprotein lipase deficiency (LPLD) and suffering from severe or multiple pancreatitis attacks despite dietary fat restrictions. The diagnosis of LPLD has to be confirmed by genetic testing. The indication is restricted to patients with detectable levels of LPL protein.	C	no	yes	yes
NovoThirteen	catridecacog	9/3/2012	Long-term prophylactic treatment of bleeding in adult and paediatric patients 6 years and above with congenital factor-XIII-A-subunit deficiency.	B	no	no	no
Revestive	teduglutide	8/30/2012	Revestive is indicated for the treatment of patients aged 1 year and above with Short Bowel Syndrome. Patients should be stable	A	no	no	yes

Medicine name	Common name	Authorisation date	Indication	ATC code (primary)	Conditional	Exceptional	Orphan
			following a period of intestinal adaptation after surgery.				
Zinforo	ceftaroline fosamil	8/23/2012	Zinforo is indicated for the treatment of the following infections in adults and children from the age of 2 months:<br><br>Complicated skin and soft tissue infections (cSSTI);<br>Community-acquired pneumonia (CAP).<br><br>Consideration should be given to official guidance on the appropriate use of antibacterial agents.	J	no	no	no
Fycompa	perampanel	7/23/2012	Fycompa is indicated for the adjunctive treatment of partial-onset seizures with or without secondarily generalised seizures in adult and adolescent patients from 12 years of age with epilepsy.Fycompa is indicated for the adjunctive treatment of primary generalised tonic-clonic seizures in adult and adolescent patients from 12 years of age with idiopathic generalised epilepsy.	N	no	no	no
Kalydeco	ivacaftor	7/23/2012	Kalydeco tablets are indicated for the treatment of patients with cystic fibrosis (CF) in patients aged 6 years and older and weighing 25 kg or more who have one of the following gating (class III) mutations in the CFTR gene: G551D, G1244E, G1349D, G178R, G551S, S1251N, S1255P, S549N, or S549R. Kalydeco is also indicated for the treatment of patients with cystic fibrosis (CF) aged 18 years and older who have an R117H mutation in the CFTR gene.	R	no	no	yes
Rienso	ferumoxytol	6/15/2012	Rienso is indicated for the intravenous treatment of iron-deficiency anaemia in adult patients	B	no	no	no

Medicine name	Common name	Authorisation date	Indication	ATC code (primary)	Conditional	Exceptional	Orphan
			with chronic kidney disease (CKD). The diagnosis of iron deficiency must be based on appropriate laboratory tests (see section 4.2).				
Pixuvri	pixantrone dimaleate	5/10/2012	Pixuvri is indicated as monotherapy for the treatment of adult patients with multiply relapsed or refractory aggressive non-Hodgkin B-cell lymphomas (NHL). The benefit of pixantrone treatment has not been established in patients when used as fifth-line or greater chemotherapy in patients who are refractory to last therapy.	L	yes	no	no
Caprelsa	vandetanib	2/17/2012	Caprelsa is indicated for the treatment of aggressive and symptomatic medullary thyroid cancer (MTC) in patients with unresectable locally advanced or metastatic disease.<br>For patients in whom re-arranged-during-transfection(RET) mutation is not known or is negative, a possible lower benefit should be taken into account before individual treatment decision.	L	yes	no	no
Zelboraf	vemurafenib	2/17/2012	Vemurafenib is indicated in monotherapy for the treatment of adult patients with BRAF-V600-mutation-positive unresectable or metastatic melanoma.	L	no	no	no
Vepacel	Influenza virus (whole virion, inactivated), containing antigen of: A/Vietnam/1203/2004 (H5N1)	2/17/2012	Active immunisation against H5N1 subtype of influenza A virus.<br>This indication is based on immunogenicity data from subjects from the age of 6 months onwards following administration of two doses of vaccine prepared with H5N1 subtype strains.<br>Vepacelshould be used in accordance with Official guidance.	J	no	no	no
Edarbi	azilsartan medoxomil	12/7/2011	Edarbi is indicated for the treatment of essential hypertension in adults.	C	no	no	no

Medicine name	Common name	Authorisation date	Indication	ATC code (primary)	Conditional	Exceptional	Orphan
Dificlir	fidaxomicin	12/5/2011	Dificlir is indicated in adults for the treatment of Clostridium difficile infections (CDI) also known as C. difficile-associated diarrhoea (CDAD).<br>Consideration should be given to official guidelines on the appropriate use of antibacterial agents.	A	no	no	no
Edurant	rilpivirine hydrochloride	11/28/2011	Edurant, in combination with other antiretroviral medicinal products, is indicated for the treatment of human immunodeficiency virus type1 (HIV1) infection in antiretroviral treatmentnave patients 12years of age and older with a viral load 100,000HIV1 RNA copies/ml.<br>As with other antiretroviral medicinal products, genotypic resistance testing should guide the use of Edurant.	J	no	no	no
Eviplera	emtricitabine / rilpivirine hydrochloride /tenofovir disoproxil fumarate	11/28/2011	Eviplera is indicated for the treatment of adults infected with human immunodeficiency virus type 1 (HIV-1) without known mutations associated with resistance to the non-nucleoside reverse transcriptase inhibitor (NNRTI) class, tenofovir or emtricitabine, and with a viral load 100,000 HIV-1 RNA copies/mL. As with other antiretroviral medicinal products, genotypic resistance testing and/or historical resistance data should guide the use of Eviplera.	J	no	no	no
Eurartesim	piperaquine tetraphosphate / dihydroartemisinin	10/27/2011	Eurartesim is indicated for the treatment of uncomplicated Plasmodium falciparum malaria in adults, children and infants 6 months and over and weighing 5 kg or more.<br>Consideration should be given to official guidance on the appropriate use of antimalarial agents.	P	no	no	no

Medicine name	Common name	Authorisation date	Indication	ATC code (primary)	Conditional	Exceptional	Orphan
Vibativ	telavancin	9/2/2011	Vibativ is indicated for the treatment of adults with nosocomial pneumoniaincluding ventilator-associated pneumonia, known or suspected to be caused by methicillin-resistant Staphylococcus aureus (MRSA).Vibativ should be used only in situations where it is known or suspected that other alternatives are not suitable.Consideration should be given to official guidance on the appropriate use of antibacterial agents.	J	no	no	no
Trajenta	linagliptin	8/24/2011	Trajenta is indicated in the treatment of type 2 diabetes mellitus to improve glycaemic control in adults: as monotherapy<br><br>in patients inadequately controlled by diet and exercise alone and for whom metformin is inappropriate due to intolerance, or contraindicated due to renal impairment.<br><br>as combination therapy<br><br>in combination with metformin when diet and exercise plus metformin alone do not provide adequate glycaemic control.<br>in combination with a sulphonylurea and metformin when diet and exercise plus dual therapy with these medicinal products do not provide adequate glycaemic control.<br>in combination with insulin with or without metformin, when this regimen alone, with diet and exercise, does not provide adequate glycaemic control.	A	no	no	no

Medicine name	Common name	Authorisation date	Indication	ATC code (primary)	Conditional	Exceptional	Orphan
Fampyra	fampridine	7/20/2011	Fampyra is indicated for the improvement of walking in adult patients with multiple sclerosis with walking disability (Expanded Disability Status Scale 4-7).	N	yes	no	no
Victrelis	boceprevir	7/18/2011	Victrelis is indicated for the treatment of chronic hepatitis-C (CHC) genotype-1 infection, in combination with peginterferon alfa and ribavirin, in adult patients with compensated liver disease who are previously untreated or who have failed previous therapy.	J	no	no	no
Benlysta	belimumab	7/13/2011	Benlysta is indicated as add-on therapy in adult patients with active, auto-antibody-positive systemic lupus erythematosus (SLE) with a high degree of disease activity (e.g. positive anti-dsDNA and low complement) despite standard therapy.	L	no	no	no
Yervoy	ipilimumab	7/13/2011	Yervoy is indicated for the treatment of advanced (unresectable or metastatic) melanoma in adults.	L	no	no	no
Bydureon	exenatide	6/17/2011	Bydureon is indicated for treatment of type-2 diabetes mellitus in combination with:<br><br>metformin;<br>sulphonylurea;<br>thiazolidinedione;<br>metformin and sulphonylurea;<br>metformin and thiazolidinedione;<br><br>in adults who have not achieved adequate glycaemic control on maximally tolerated doses of these oral therapies.	A	no	no	no
Nulojix	belatacept	6/17/2011	Nulojix, in combination with corticosteroids and a mycophenolic acid (MPA), is indicated for prophylaxis of graft rejectionin adults receiving a renal transplant (see	L	no	no	no

Medicine name	Common name	Authorisation date	Indication	ATC code (primary)	Conditional	Exceptional	Orphan
			section 5.1 for data on renal function). It is recommended to add an interleukin (IL)-2-receptor antagonist for induction therapy to this belatacept-based regimen.				
Cinryze	C1 inhibitor (human)	6/15/2011	Treatment and pre-procedure prevention of angioedema attacks in adults and adolescents with hereditary angioedema (HAE). Routine prevention of angioedema attacks in adults and adolescents with severe and recurrent attacks of hereditary angioedema (HAE), who are intolerant to or insufficiently protected by oral prevention treatments, or patients who are inadequately managed with repeated acute treatment.	B	no	no	no
Trobalt	retigabine	3/28/2011	Trobalt is indicated as adjunctive treatment of drug-resistant partial-onset seizures with or without secondary generalisation in patients aged 18 years or older with epilepsy, where other appropriate drug combinations have proved inadequate or have not been tolerated.	N	no	no	no
Halaven	eribulin	3/17/2011	Halaven monotherapy is indicated for the treatment of patients with locally advanced or metastatic breast cancer who have progressed after at least two chemotherapeutic regimens for advanced disease (see section 5.1). Prior therapy should have included an anthracycline and a taxane unless patients were not suitable for these treatments. Halaven is indicated for the treatment of adult patients with unresectable liposarcoma who have received prior anthracycline containing therapy (unless unsuitable) for advanced or	L	no	no	no

Medicine name	Common name	Authorisation date	Indication	ATC code (primary)	Conditional	Exceptional	Orphan
			metastatic disease (see section 5.1).				
Gilenya	fingolimod hydrochloride	3/17/2011	Gilenya is indicated as single disease-modifying therapy in highly active relapsing-remitting multiple sclerosis for the following adult patient groups:<br><br>patients patients with highly active disease despite a full and adequate course of treatment with at least one disease modifying therapy (for exceptions and information about washout periods see sections 4.4 and 5.1).<br><br>or:<br><br>patients with rapidly evolving severe relapsing remitting multiple sclerosis defined bytwo or more disabling relapses in one year, and withone or more gadolinium-enhancing lesions on brain MRI or a significant increase in T2 lesion load as compared to a previous recent MRI.	L	no	no	no
Xeplion	paliperidone palmitate	3/4/2011	Xeplion is indicated for maintenance treatment of schizophrenia in adult patients stabilised with paliperidone or risperidone.In selected adult patients with schizophrenia and previous responsiveness to oral paliperidone or risperidone, Xeplion may be used without prior stabilisation with oral treatment if psychotic symptoms are mild to moderate and a long-acting injectable treatment is needed.	N	no	no	no
Pumarix	pandemic influenza vaccine (H5N1) (split virion, inactivated, adjuvanted)	3/4/2011	Prophylaxis of influenza in an officially declared pandemic situation.<br>Pandemic-influenza vaccine should be used in accordance with official	J	no	yes	no

Medicine name	Common name	Authorisation date	Indication	ATC code (primary)	Conditional	Exceptional	Orphan
			guidance.				
Brilique	ticagrelor	12/3/2010	Brilique, co administered with acetylsalicylic acid (ASA), is indicated for the prevention of atherothrombotic events in adult patients with<br>acute coronary syndromes (ACS) or a history of myocardial infarction (MI) and a high risk of developing an atherothrombotic event<br>Brilique, co-administered with acetyl salicylic acid (ASA), is indicated for the prevention of atherothrombotic events in adult patients with a history of myocardial infarction (MI occurred at least one year ago) and a high risk of developing an atherothrombotic event.	B	no	no	no
Aflunov	influenza virus surface antigens (haemagglutinin and neuraminidase) of strain: A/turkey/Turkey/1/05 (H5N1)-like strain (NIBRG-23)	11/29/2010	Active immunisation against H5N1 subtype of influenza-A virus.<br>This indication is based on immunogenicity data from healthy subjects from the age of 18 years onwards following administration of two doses of the vaccine containing A/turkey/Turkey/1/05 (H5N1)-like strain.Aflunov should be used in accordance with official recommendations.	J	no	no	no
Ruconest	recombinant human c1-inhibitor	10/28/2010	Ruconest is indicated for treatment of acute angioedema attacks in adults with hereditary angioedema (HAE) due to C1-esterase-inhibitor deficiency.	B	no	no	no
Rapiscan	regadenoson	9/6/2010	This medicinal product is for diagnostic use only.<br>Rapiscan is a selective coronary vasodilator for use as a pharmacological stress agent for radionuclide myocardial perfusion imaging (MPI) in adult patients unable to undergo adequate	C	no	no	no

Medicine name	Common name	Authorisation date	Indication	ATC code (primary)	Conditional	Exceptional	Orphan
			exercise stress.				
Sycrest	asenapine maleate	9/1/2010	Sycrest is indicated for the treatment of moderate to severe manic episodes associated with bipolar I disorder in adults.	N	no	no	no
Vpriv	velaglucerase alfa	8/26/2010	Vpriv is indicated for long-term enzyme-replacement therapy (ERT) in patients with type-1 Gaucher disease.	A	no	no	yes
Votrient	pazopanib	6/14/2010	Renal-cell carcinoma (RCC)<br>Votrient is indicated in adults for the first-line treatment of advanced renal-cell carcinoma (RCC) and for patients who have received prior cytokine therapy for advanced disease.<br>Soft-tissue sarcoma (STS)<br>Votrient is indicated for the treatment of adult patients with selective subtypes of advanced soft-tissue sarcoma (STS) who have received prior chemotherapy for metastatic disease or who have progressed within 12 months after (neo)adjuvant therapy.<br>Efficacy and safety have only been established in certain STS histological tumour subtypes.	L	no	no	no
Humenza	split influenza virus, inactivated, containing antigen*: A/California/7/2009 (H1N1)v like strain (X-179A)<br>*propagated in eggs.	6/8/2010	Prophylaxis of influenza in an officially declared pandemic situation.Pandemic influenza vaccine should be used in accordance with Official Guidance.	J	yes	no	no
Prolia	denosumab	5/26/2010	Treatment of osteoporosis in postmenopausal women and in men at increased risk of fractures. In postmenopausal women Prolia significantly reduces the risk of vertebral, non vertebral and hip fractures.Treatment of bone loss	M	no	no	no

Medicine name	Common name	Authorisation date	Indication	ATC code (primary)	Conditional	Exceptional	Orphan
			associated with hormone ablation in men with prostate cancer at increased risk of fractures. In men with prostate cancer receiving hormone ablation, Prolia significantly reduces the risk of vertebral fractures.				
Arepanrix	split influenza virus, inactivated, containing antigen* : A/California/7/2009 (H1N1)v like strain (X-179A)<br>* produced in eggs	3/23/2010	Prophylaxis of influenza in an officially declared pandemic situation. Pandemic influenza vaccine should be used in accordance with official guidance	J	yes	no	no
Menveo	meningococcal group A, C, W-135 and Y conjugate vaccine	3/15/2010	Prefilled syringe<br>Menveo is indicated for active immunisation of adolescents (from 11 years of age) and adults at risk of exposure to Neisseria meningitidis groups A, C, W135 and Y, to prevent invasive disease.<br>The use of this vaccine should be in accordance with official recommendations.<br>Vials<br>Menveo is indicated for active immunisation of children (fromtwo years of age), adolescents and adults at risk of exposure to Neisseria meningitidis groups A, C, W135 and Y, to prevent invasive disease.<br>The use of this vaccine should be in accordance with official recommendations.	J	no	no	no
Revolade	eltrombopag olamine	3/11/2010	Revolade is indicated for chronic immune (idiopathic) thrombocytopenic purpura (ITP) patients aged 1 year and above who are refractory to other treatments (e.g. corticosteroids, immunoglobulins) (see sections 4.2 and 5.1).	B	no	no	no

Medicine name	Common name	Authorisation date	Indication	ATC code (primary)	Conditional	Exceptional	Orphan
			Revolade is indicated in adult patients with chronic hepatitis C virus (HCV) infection for the treatment of thrombocytopenia, where the degree of thrombocytopenia is the main factor preventing the initiation or limiting the ability to maintain optimal interferon-based therapy (see sections 4.4 and 5.1). Revolade is indicated in adult patients with acquired severe aplastic anaemia (SAA) who were either refractory to prior immunosuppressive therapy or heavily pretreated and are unsuitable for haematopoietic stem cell transplantation (see section 5.1).				
Harvoni	ledispavir 90 mg / sofosbuvir 400 mg	11/17/2014	Harvoni is indicated for the treatment of chronic hepatitis C (CHC) in adults.	J	no	no	no
Velphoro	mixture of polynuclear iron(III)-oxyhydroxide, sucrose and starches	8/26/2014	Velphoro is indicated for the control of serum phosphorus levels in adult chronic kidney disease (CKD) patients on haemodialysis (HD) or peritoneal dialysis (PD). Velphoro should be used within the context of a multiple therapeutic approach, which could include calcium supplement, 1,25-dihydroxy vitamin D3 or one of its analogues, or calcimimetics to control the development of renal bone disease.	V	no	no	no
Deltyba	delamanid	4/28/2014	Deltyba is indicated for use as part of an appropriate combination regimen for pulmonary multi-drug resistant tuberculosis (MDR-TB) in adult patients when an effective treatment regimen cannot otherwise be composed for reasons of resistance or tolerability. Consideration should be given to official guidance on the appropriate	J	yes	no	yes

Medicine name	Common name	Authorisation date	Indication	ATC code (primary)	Conditional	Exceptional	Orphan
			use of antibacterial agents.				
Vimizim	recombinant human n-acetylgalactosamine-6-sulfatase (rhgalns)	4/28/2014	Vimizim is indicated for the treatment of mucopolysaccharidosis, type IVA (Morquio A Syndrome, MPS IVA) in patients of all ages.	A	no	no	yes
Hemangiol	propranolol hydrochloride	4/23/2014	Hemangiol is indicated in the treatment of proliferating infantile haemangioma requiring systemic therapy:- Life- or function-threatening haemangioma,- Ulcerated haemangioma with pain and/or lack of response to simple wound care measures,- Haemangioma with a risk of permanent scars or disfigurement.It is to be initiated in infants aged 5 weeks to 5 months	C	no	no	no
Sirturo	bedaquiline fumarate	3/5/2014	Indicated for use as part of an appropriate combination regimen for pulmonary multidrug resistant tuberculosis (MDR TB) in adult patients when an effective treatment regimen cannot otherwise be composed for reasons of resistance or tolerability.<br>Consideration should be given to official guidance on the appropriate use of antibacterial agents.	J	yes	no	yes
Fluenz Tetra	influenza virus type A, H1N1 / influenza virus type A, H3N2 / influenza virus type B (Victoria lineage) / influenza virus, type B (Yamagata lineage)	12/4/2013	Prophylaxis of influenza in children and adolescents 24months to less than 18years of age. The use of Fluenz Tetra should be based on official recommendations.	J	no	no	no
Vitekta	elvitegravir	11/13/2013	Vitekta co-administered with a ritonavir-boosted protease inhibitor and with other antiretroviral agents, is indicated for the treatment of human-immunodeficiency-virus-1 (HIV-1) infection in adults who are infected with HIV-1 without known	J	no	no	no

Medicine name	Common name	Authorisation date	Indication	ATC code (primary)	Conditional	Exceptional	Orphan
			mutations associated with resistance to elvitegravir.				
Tybost	cobicistat on silicon dioxide	9/19/2013	Tybost is indicated as a pharmacokinetic enhancer of atazanavir 300 mg once daily or darunavir 800 mg once daily as part of antiretroviral combination therapy in human-immunodeficiency-virus-1 (HIV-1)-infected adults.	V	no	no	no
Imvanex	modified vaccinia Ankara - Bavarian Nordic (MVA-BN) virus	7/31/2013	Active immunisation against smallpox infection and disease in adults.	J	no	yes	no
Stribild	elvitegravir / cobicistat / emtricitabine / tenofovir disoproxil fumarate	5/24/2013	Treatment of human immunodeficiency virus 1 (HIV 1) infection in adults aged 18 years and over who are antiretroviral treatment-nave or are infected with HIV 1 without known mutations associated with resistance to any of the three antiretroviral agents in Stribild.	J	no	no	no
HyQvia	human normal immunoglobulin	5/16/2013	Replacement therapy in adults, children and adolescents (0-18 years) in:<br><br>Primary immunodeficiency syndromes with impaired antibody production (see section4.4).<br>Hypogammaglobulinaemia and recurrent bacterial infections in patients with chronic lymphocytic leukaemia (CLL), in whom prophylactic antibiotics have failed or are contraindicated.<br>Hypogammaglobulinaemia and recurrent bacterial infections in multiple myeloma (MM) patients.<br>Hypogammaglobulinaemia in patients pre and postallogeneic hematopoietic stem cell transplantation (HSCT).	J	no	no	no
Hexyon	diphtheria toxoid/ tetanus toxoid/ two-component acellular	4/17/2013	Primary and booster vaccination of infants and toddlers from six weeks to 24 months of age against	J	no	no	no

Medicine name	Common name	Authorisation date	Indication	ATC code (primary)	Conditional	Exceptional	Orphan
	pertussis (pertussis toxoidand filamentous haemagglutinin)/ inactivated poliomyelitis virus types 1, 2 and 3 /Haemophilus influenzae type-b polysaccharide (polyribosylribitol phosphate) conjugated to tetanus protein /hepatitis-B surface antigen		diphtheria, tetanus, pertussis, hepatitis B, poliomyelitis and invasive diseases caused by Haemophilusinfluenzae type b (Hib).				
Hexacima	diphtheria toxoid /tetanus toxoid / two-component acellular pertussis(pertussis toxoidand filamentous haemagglutinin) /inactivated poliomyelitis virus types 1,2 and 3 /Haemophilus influenzae type-b polysaccharide (polyribosylribitol phosphate) conjugated to tetanus protein / hepatitis-B surface antigen	4/17/2013	Primary and booster vaccination of infants and toddlers from six weeks to 24 months of age against diphtheria, tetanus, pertussis, hepatitis B, poliomyelitis and invasive diseases caused by Haemophilusinfluenzae type-b (Hib).	J	no	no	no
Bexsero	recombinant Neisseria meningitidis group-B NHBA fusion protein /recombinant Neisseria meningitidis group-B NadA protein /recombinant Neisseria meningitidis group B fHbp fusion protein /outer membrane vesiclesfrom Neisseria meningitidis group-B strain NZ98 / 254 measured as amount of total protein containing the PorA P1.4	1/14/2013	Active immunisation against invasive disease caused by Neisseria meningitidis serogroup-B strains.	J	no	no	no

Medicine name	Common name	Authorisation date	Indication	ATC code (primary)	Conditional	Exceptional	Orphan
Forxiga	dapagliflozin propanediol monohydrate	11/12/2012	Forxiga is indicated in adults aged 18 years and older with type-2 diabetes mellitus to improve glycaemic control as:<br><br>monotherapy when diet and exercise alone do not provide adequate glycaemic control in patients for whom use of metformin is considered inappropriate due to intolerance;<br>add-on combination therapy in combination with other glucose-lowering medicinal products including insulin, when these, together with diet and exercise, do not provide adequate glycaemic control.	A	no	no	no
Nimenrix	Neisseria meningitidis serogroup A polysaccharide conjugated to tetanus toxoid / Neisseria meningitidis serogroup C polysaccharide conjugated to tetanus toxoid /Neisseria meningitidis serogroup W-135 polysaccharide conjugated to tetanus toxoid / Neisseria meningitidis serogroup Y polysaccharide conjugated to tetanus toxoid	4/20/2012	Nimenrix is indicated for active immunisation of individuals from the age of 12 months and above against invasive meningococcal diseases caused by Neisseria meningitidis serogroups A, C, W-135 and Y.	J	no	no	no
Bronchitol	mannitol	4/13/2012	Bronchitol is indicated for the treatment of cystic fibrosis (CF) in adults aged 18 years and above as an add-on therapy to best standard of care.	R	no	no	yes
Colobreathe	colistimethate sodium	2/13/2012	Colobreathe is indicated for the management of chronic pulmonary infections due to Pseudomonas	J	no	no	no

Medicine name	Common name	Authorisation date	Indication	ATC code (primary)	Conditional	Exceptional	Orphan
			aeruginosa in patients with cystic fibrosis (CF) agedsix years and older.<br>Consideration should be given to official guidance on the appropriate use of antibacterial agents.				
Ipreziv	azilsartan medoxomil	12/7/2011	Ipreziv is indicated for the treatment of essential hypertension in adults.	C	no	no	no
Ioa	nomegestrol acetate / estradiol	11/16/2011	Oral contraception.	G	no	no	no
Incivo	telaprevir	9/19/2011	Incivo, in combination with peginterferon alfa and ribavirin, is indicated for the treatment of genotype-1 chronic hepatitis C in adult patients with compensated liver disease (including cirrhosis):<br><br>who are treatment nave;<br>who have previously been treated with interferon alfa (pegylated or non-pegylated) alone or in combination with ribavirin, including relapsers, partial responders and null responders.	J	no	no	no
Votubia	everolimus	9/2/2011	Renal angiomyolipoma associated with tuberous sclerosis complex (TSC)<br>Votubia is indicated for the treatment of adult patients with renal angiomyolipoma associated with tuberous sclerosis complex (TSC) who are at risk of complications (based on factors such as tumour size or presence of aneurysm, or presence of multiple or bilateral tumours) but who do not require immediate surgery.<br>The evidence is based on analysis of change in sum of angiomyolipoma volume.<br>Subependymal giant cell astrocytoma (SEGA) associated with tuberous sclerosis complex (TSC)	L	no	no	yes

Medicine name	Common name	Authorisation date	Indication	ATC code (primary)	Conditional	Exceptional	Orphan
			Votubia is indicated for the treatment of patients with subependymal giant cell astrocytoma (SEGA) associated with tuberous sclerosis complex (TSC) who require therapeutic intervention but are not amenable to surgery.<br>The evidence is based on analysis of change in SEGA volume. Further clinical benefit, such as improvement in diseaserelated symptoms, has not been demonstrated.				
Tobi Podhaler	tobramycin	7/20/2011	Tobi Podhaler is indicated for the suppressive therapy of chronic pulmonary infection due to Pseudomonas aeruginosa in adults and children aged 6 years and older with cystic fibrosis. See sections 4.4 and 5.1 regarding data in different age groups.Consideration should be given to official guidance on the appropriate use of antibacterial agents.	J	no	no	yes
Xgeva	denosumab	7/13/2011	Prevention of skeletal related events (pathological fracture, radiation to bone, spinal cord compression or surgery to bone) in adults with bone metastases from solid tumours.<br>Treatment of adults and skeletally mature adolescents with giant cell tumour of bone that is unresectable or where surgical resection is likely to result in severe morbidity.	M	no	no	no
Eliquis	apixaban	5/18/2011	Prevention of venous thromboembolic events (VTE) in adult patients who have undergone elective hip- or knee-replacement surgery.	B	no	no	no
Fluenz	Reassortant influenza virus (live attenuated) of the following strains: A/California/7/2009 (H1N1)pdm09 like	1/27/2011	Prophylaxis of influenza in individuals 24 months to less than 18 years of age.<br>The use of Fluenz should be based on official recommendations.	J	no	no	no

Medicine name	Common name	Authorisation date	Indication	ATC code (primary)	Conditional	Exceptional	Orphan
	strain, A/Victoria/361/2011 (H3N2) like strain, B/Massachusetts/2/2012 like strain						
Possia	ticagrelor	12/3/2010	Possia, co-administered with acetylsalicylic acid (ASA), is indicated for the prevention of atherothrombotic events in adult patients with acute coronary syndromes (unstable angina, non-ST-elevation myocardial infarction [NSTEMI] or ST-elevation myocardial infarction [STEMI]); including patients managed medically, and those who are managed with percutaneous coronary intervention (PCI) or coronary artery by-pass grafting (CABG).	B	no	no	no
Prepandemic influenza vaccine (H5N1) (surface antigen, inactivated, adjuvanted) Novartis Vaccines and Diagnostics	influenza virus surface antigens (haemagglutinin and neuraminidase) of strain A/Viet Nam/1194/2004 (H5N1)	11/29/2010	Active immunisation against H5N1 subtype of Influenza A virus.This indication is based on immunogenicity data from healthy subjects from the age of 18 years onwards following administration of two doses of the vaccine containing A/Vietnam/1194/2004 (H5N1)-like strain.Prepandemic influenza vaccine (H5N1) Novartis Vaccines and Diagnostic should be used in accordance with official recommendations.	J	no	no	no
Elonva	corifollitropin alfa	1/25/2010	Controlled ovarian stimulation in combination with a gonadotrophin-releasing-hormone antagonist for the development of multiple follicles in women participating in an assisted-reproductive-technology programme.	G	no	no	no
