# Supplementary material for: Delays in completion and results reporting of clinical trials under the Paediatric Regulation in the European Union: A cohort study
Source: PLoS Med. 2018 Mar 1;15(3):e1002520. doi: 10.1371/journal.pmed.1002520 (PMC5832187; doi:10.1371/journal.pmed.1002520)
Supplement: S1 Protocol — (PDF) [file pmed.1002520.s002.pdf]

ANNEX I

SUMMARY PROTOCOL FOR PROPOSED STUDY ON PEDIATRIC REGULATION

**Primary Investigators:**	Thomas Hwang, Florence Bourgeois
**Protocol Title:**	Delays and Discontinuation of Pediatric Studies for New Medicines Approved by the European Medicines Agency (EMA)
**Study Objective:**	To assess the frequency, duration, and reasons for delays and non-completion of pediatric study requirements for new medicines authorized by the EMA
**Study Design:**	A retrospective analysis of publicly available information on new drugs authorized by the EMA and associated pediatric study requirements.
**Study Population:**	New drugs that were approved by the EMA between 2010 and 2014 (with follow-up through 2017) with required pediatric studies.
**Sample Size:**	There are approximately ~100 new medicines authorized by EMA during our study period. Therefore, we estimate that our final cohort will include 200-300 pediatric trials (assuming 2-3 pediatric trials per medicine).
**Study Endpoints:**	• Median expected time to study completion in the original Pediatric Investigation Plan (PIP) and as of date of final follow-up (to be determined)<br>• Rates of completion, discontinuation, publication, and results reporting of pediatric trials in the PIP, stratified by trial type (efficacy, safety, PK/PD)<br>• Frequency of deferrals of pediatric trials and duration of deferral and any extensions<br>• Frequency of modifications to PIPs and types of modifications (e.g., sample size reduction, study endpoints, completion date)
**Statistical Methods:**	Descriptive statistics will be used to characterize the pediatric trials comprising our final analysis datatset for the identified new medicines authorized by the EMA with pediatric study requirements.<br><br>Fisher’s exact test and non-parametric tests will be used in unadjusted, univariable analyses.<br><br>Multivariable Cox proportional hazards will be used to identify predictors of successful pediatric trial completion
