# Supplementary material for: Delays in completion and results reporting of clinical trials under the Paediatric Regulation in the European Union: A cohort study
Source: PLoS Med. 2018 Mar 1;15(3):e1002520. doi: 10.1371/journal.pmed.1002520 (PMC5832187; doi:10.1371/journal.pmed.1002520)
Supplement: S1 Table — Results from multivariable logistic regression models for odds of delay to study completion (i.e., extension granted to planned completion date). Antineoplastic (ATC code L) also includes immunomodulatory agents. aWorld Health Organization ATC therapeutic area. bOmitted due to collinearity. MA, marketing authorisation. (DOCX) [file pmed.1002520.s003.docx]

**S1 Table.** Results from Multivariable Logistic Regression Models of Study Delay

| **Characteristic** | **Adjusted Odds Ratio (aOR)** | **95% Confidence Interval (CI)** | *P value* |
| --- | --- | --- | --- |
| PIP opinion year |  |  |  |
| 2008 | 1 [Reference] |  |  |
| 2009 | 1.01 | (0.26-3.87) | 0.99 |
| 2010 | 0.90 | (0.19-4.28) | 0.89 |
| 2011 | 1.59 | (0.41-6.14) | 0.50 |
| 2012 | 0.42 | (0.09-1.92) | 0.27 |
| 2013 | –^b^ | –^b^ | –^b^ |
| Therapeutic area ^a^ |  |  |  |
| Alimentary and metabolism | 1 [Reference] |  |  |
| Blood | 0.25 | (0.07-0.88) | 0.03 |
| Cardiovascular | 0.66 | (0.13-3.42) | 0.62 |
| Genitourinary | 0.05 | (0.00-0.72) | 0.03 |
| Anti-infective | 0.82 | (0.22-3.01) | 0.76 |
| Antineoplastic | 1.26 | (0.29-5.40) | 0.76 |
| Neurologic | 0.58 | (0.15-2.20) | 0.42 |
| Respiratory | 0.30 | (0.07-1.34) | 0.12 |
| Musculoskeletal and others | 1.14 | (0.26-5.08) | 0.86 |
| Planned completion after MA |  |  |  |
| Yes | 1.17 | (0.59-2.35) | 0.65 |
| No | 1 [Reference] |  |  |
| Orphan drug status |  |  |  |
| Yes | 0.44 | (0.18-1.05) | 0.06 |
| No | 1 [Reference] |  |  |
| Study type |  |  |  |
| PK/PD only | 1 [Reference] |  |  |
| Primarily efficacy | 1.14 | (0.50-2.60) | 0.75 |
| Efficacy and safety | 0.58 | (0.14-2.51) | 0.47 |
| Primarily safety | 0.95 | (0.40-2.28) | 0.91 |
| Total modifications | 2.31 | (1.18-4.51) | 0.02 |
| Modification type |  |  |  |
| Study size | 0.86 | (0.29-2.54) | 0.79 |
| Endpoints | 0.95 | (0.27-3.38) | 0.93 |
| Population | 0.59 | (0.21-1.63) | 0.31 |
| Statistical methods | 0.58 | (0.18-1.87) | 0.37 |
| Treatment duration | 0.29 | (0.11-0.76) | 0.01 |
| Other changes | –^b^ | –^b^ | –^b^ |
| Planned study enrolment (N) | 1.00 | (1.00-1.00) | 0.91 |
