# Supplementary material for: Delays in completion and results reporting of clinical trials under the Paediatric Regulation in the European Union: A cohort study
Source: PLoS Med. 2018 Mar 1;15(3):e1002520. doi: 10.1371/journal.pmed.1002520 (PMC5832187; doi:10.1371/journal.pmed.1002520)
Supplement: S2 Table — Hazard ratios (HRs) and CIs are from multivariable Cox regression models for trial completion of all trials (i.e., including trials required for medicines authorised for use in any paediatric age group) and of efficacy and safety trials only (efficacy/safety; i.e., excluding PK/PD studies). Antineoplastic (ATC code L) also includes immunomodulatory agents. aWorld Health Organization ATC therapeutic area. bOmitted due to collinearity. MA, marketing authorisation; PK/PD, pharmacokinetic/pharmacodynamics. (DOCX) [file pmed.1002520.s004.docx]

**S2 Table.** Results from Multivariable Cox Regression Models of Trial Completion

| **Characteristic** | **HR (95% CI)**  ***All Trials*** | *P value* |  | **HR (95% CI)**  ***Efficacy/Safety*** | *P value* |
| --- | --- | --- | --- | --- | --- |
| PIP opinion year |  |  |  |  |  |
| 2008 | 1 [Reference] |  |  | 1 [Reference] |  |
| 2009 | 0.62 (0.24-1.56) | 0.31 |  | 0.55 (0.20-1.57) | 0.27 |
| 2010 | 1.26 (0.50-3.22) | 0.62 |  | 0.76 (0.30-1.93) | 0.57 |
| 2011 | 1.05 (0.40-2.75) | 0.92 |  | 0.61 (0.18-1.99) | 0.41 |
| 2012 | 0.72 (0.16-3.18) | 0.66 |  | 0.41 (0.10-1.72) | 0.22 |
| 2013 | –^b^ | –^b^ |  | –^b^ | –^b^ |
| Therapeutic area ^a^ |  |  |  |  |  |
| Alimentary and metabolism | 1 [Reference] |  |  | 1 [Reference] |  |
| Blood | 1.86 (0.79-4.34) | 0.15 |  | 1.63 (0.52-5.09) | 0.40 |
| Cardiovascular | 0.56 (0.18-1.72) | 0.31 |  | 0.30 (0.06-1.54) | 0.15 |
| Genitourinary | 0.75 (0.29-1.93) | 0.55 |  | –^b^ | –^b^ |
| Anti-infective | 1.55 (0.63-3.82) | 0.34 |  | 2.11 (0.66-6.73) | 0.21 |
| Antineoplastic | 0.48 (0.18-1.24) | 0.13 |  | 0.19 (0.05-0.74) | 0.02 |
| Neurologic | 0.83 (0.29-2.33) | 0.72 |  | 1.14 (0.34-3.83) | 0.83 |
| Respiratory | 2.25 (0.85-5.92) | 0.10 |  | 1.80 (0.58-5.57) | 0.31 |
| Musculoskeletal and others | 0.73 (0.07-7.91) | 0.80 |  | 0.51 (0.05-5.81) | 0.59 |
| Planned completion after MA |  |  |  |  |  |
| Yes | 0.11 (0.06-0.19) | <0.001 |  | 0.09 (0.05-0.18) | <0.001 |
| No | 1 [Reference] |  |  | 1 [Reference] |  |
| Orphan drug status |  |  |  |  |  |
| Yes | 0.88 (0.50-1.53) | 0.64 |  | 0.92 (0.49-1.71) | 0.79 |
| No | 1 [Reference] |  |  | 1 [Reference] |  |
| Study type |  |  |  |  |  |
| PK/PD only | 1 [Reference] |  |  |  |  |
| Primarily efficacy | 0.81 (0.39-1.68) | 0.56 |  | 1 [Reference] |  |
| Efficacy and safety | 0.98 (0.37-2.58) | 0.97 |  | 1.26 (0.54-2.94) | 0.59 |
| Primarily safety | 0.76 (0.39-1.50) | 0.43 |  | 0.77 (0.41-1.44) | 0.41 |
| Planned study enrolment (N) | 1.00 (1.00-1.00) | 0.12 |  | 1.00 (1.00-1.00) | 0.85 |
