# Supplementary material for: Delays in completion and results reporting of clinical trials under the Paediatric Regulation in the European Union: A cohort study
Source: PLoS Med. 2018 Mar 1;15(3):e1002520. doi: 10.1371/journal.pmed.1002520 (PMC5832187; doi:10.1371/journal.pmed.1002520)
Supplement: S3 Table — The study cohort of paediatric trials relates to 122 new medicines that were centrally authorised by the EMA between 2010 and 2014 with paediatric requirements, of which 86 were initially authorised for use in adults only (see Methods for details on cohort construction and definitions). PIP, paediatric investigation plan; PK/PD, pharmacokinetic/pharmacodynamics. (DOCX) [file pmed.1002520.s005.docx]

**S3 Table.** Characteristics of Paediatric Trials for New Medicines Initially Authorised for Adults Only in 2010-2014

|  | **Authorised for adults only, no. (%)** |
| --- | --- |
| Total paediatric trials, n | 203 |
| Therapeutic area |  |
| Alimentary and metabolism | 32 (15.8%) |
| Blood | 18 (8.9%) |
| Cardiovascular | 12 (5.9%) |
| Genitourinary | 9 (4.4%) |
| Anti-infective | 29 (14.3%) |
| Antineoplastic and immunomodulatory | 44 (21.7%) |
| Neurologic | 41 (20.2%) |
| Respiratory | 2 (1.0%) |
| Musculoskeletal and others | 16 (7.9%) |
| PIP opinion year |  |
| 2008 | 30 (14.8%) |
| 2009 | 42 (20.7%) |
| 2010 | 40 (19.7%) |
| 2011 | 49 (24.1%) |
| 2012 | 35 (17.2%) |
| 2013 | 7 (3.5%) |
| Study type |  |
| PK/PD only | 41 (20.2%) |
| Primarily efficacy | 115 (56.7%) |
| Efficacy and safety | 10 (4.9%) |
| Primarily safety | 37 (18.2%) |
| Planned completion after MA |  |
| Yes | 179 (88.2%) |
| No | 24 (11.8%) |
| Any extension of completion date |  |
| Yes | 104 (51.2%) |
| No | 99 (48.8%) |
| Any modification (excl. extensions) |  |
| Yes | 122 (60.1%) |
| No | 81 (39.9%) |
| Orphan drug status |  |
| Yes | 23 (11.3%) |
| No | 180 (88.7%) |
